# Comparing self and informant ratings of the Mild Behavioral Impairment‐Checklist in non‐demented older adults from a memory clinic‐based Czech Brain Aging Study

**DOI:** 10.1002/alz70857_105484

**Published:** 2025-12-25

**Authors:** Monika Krejci, Veronika Matuskova, Toni T Saari, Hana Horakova, Sarka Borovska, Jan Laczó, Jakub Hort, Martin Vyhnalek

**Affiliations:** ^1^ Charles University, Faculty of Arts, Prague, Czech Republic; ^2^ Charles University, Second Faculty of Medicine and Motol University Hospital, Prague, Czech Republic; ^3^ University of Helsinki, Helsinki, Finland

## Abstract

**Background:**

In individuals with cognitive deficit, neuropsychiatric symptoms (NPS) are typically assessed from the perspective of an informant. However, in the early stages of neurodegenerative diseases, patients' insight may still be preserved and their perceptions can provide valuable information. Comparison of these assessments in previous studies indicated differences in their perspectives. This study analysed differences in self‐ and informant‐rated NPS using the Mild Behavioral Impairment Checklist (MBI‐C) and examined their association with the degree of cognitive deficit.

**Method:**

The study included 127 non‐demented older adults with cognitive complaints and their informants recruited from a memory clinic‐based Czech Brain Aging Study. Participants were divided into two groups based on comprehensive neuropsychological assessment: subjective cognitive decline (SCD; *N* = 38) and mild cognitive impairment (MCI; *N* = 89). A control group consisted of 26 cognitively normal individuals with no clinically significant cognitive complaints. Both participants and their informants completed the Czech version of the MBI‐C. The differences between self and informant MBI‐C total and domain scores were examined using Wilcoxon signed‐rank test and their associations were analyzed using Spearman correlations. Further, we explored the association between MBI‐C scores and participants' cognition using regression analyses controlled for age, sex and education.

**Result:**

Both self and informant MBI‐C scores were higher in SCD and MCI groups compared to controls in most domains (*p*'s < .05), whereas MCI group had higher scores than SCD only in informant‐rated total score and decreased motivation (*p*'s < .05). Correlations between self and informant ratings were weak to moderate (r_s_ = .23–.55, *p*'s < .005). In the MCI group, informants reported more severe impulse dyscontrol symptoms than participants (*p* < .05), while no significant differences were observed in the SCD group. Informant ratings were associated more strongly with objective cognitive deficit, especially memory and executive functions (*p*'s < .05), whereas self‐ratings were less predictive of cognitive performance.

**Conclusion:**

This study underscores the importance of both self‐ and informant‐rated NPS assessments. Informants provided insights into the relationship between NPS and cognitive deficits, while self‐report can provide information on subjective experiences of these symptoms. Combining both perspectives provides complementary information beneficial for the early diagnosis of neurodegenerative diseases.

